# Insights into the multifunctionality of viral glycoproteins F and HN in the lifecycle and pathogenesis of Newcastle disease virus: a systematic review

**DOI:** 10.1186/s13567-025-01647-0

**Published:** 2025-11-05

**Authors:** Si Ma, Rongjing Xia, Wenjie Wu, Zhiqiang Duan

**Affiliations:** 1 Key Laboratory of Animal Genetics, Breeding and Reproduction in The Plateau Mountainous Region, Ministry of Education, Guizhou University, Guiyang, 550025 China; 2 College of Animal Science, Guizhou University, Guiyang, 550025 China

**Keywords:** Newcastle disease virus, F glycoprotein, HN glycoprotein, broad-spectrum antibody, antiviral drug

## Abstract

Newcastle disease virus (NDV) is a representative paramyxovirus that usually causes severe infections and substantial economic losses to the global poultry industry. Over the years, NDV has attracted widespread attention as a promising oncolytic virotherapy agent and vector vaccine against many pathogens and an important prototype for elucidating the replication and pathogenesis of other paramyxoviruses. The F and HN glycoproteins are two kinds of glycosylated transmembrane proteins located on the virion envelope that play multiple roles in the virulence, infection, replication, and pathogenicity of NDV. In view of the ability to induce neutralizing and protective antibodies and the similarity in the structural features of the F and HN glycoproteins of NDV and other paramyxoviruses, investigating their structures and functions is beneficial for understanding the viral lifecycle and pathogenesis and developing more effective broad-spectrum antibodies or antiviral drugs against viral infection. This systematic review aims to summarize the structural features and membrane fusion mechanism of the F and HN glycoproteins and their relationships with viral virulence, pathogenic phenotype and thermostability, coupled with the crucial roles of F/HN-host protein/compound interactions in the infection, replication, and pathogenicity of NDV. Additionally, this review also highlights the importance of technologies such as protein‒protein interactome analysis, single-particle cryo-electron microscopy, genome-wide CRISPR/Cas9 library screening, and computational structural biology for providing novel viewpoints on the lifecycle and pathogenesis of NDV and related paramyxoviruses and valuable reference information for the future development of efficient treatment strategies targeting viral glycoproteins.

## Introduction

Newcastle disease (ND), caused by Newcastle disease virus (NDV) infection, is among the most highly contagious and economically important avian diseases and usually leads to substantial economic losses in the global poultry industry [1]. NDV, previously known as avian paramyxovirus 1 (APMV-1), belongs to the genus *Orthoavulavirus* within the *Paramyxoviridae* family [2]. NDV strains (NDVs) can be classified into two classes, Class I and Class II, of which Class I NDVs, with a genome length of 15,198 nucleotides (nt), are composed of a single genotype (1) and are largely isolated from wild birds, whereas Class II NDVs, with a genome size category of 15 186 or 15 192 nt, are composed of 20 genotypes (I-XXI) and are generally isolated from various bird species [3, 4]. In addition, NDVs can be categorized into three pathogenic phenotypes: velogenic (high virulence), mesogenic (moderate virulence), and lentogenic (low virulence). There is a consensus that Class I NDVs are typically avirulent, whereas Class II NDVs show a wide variety of virulence and pathotypes at the early stages [5]. However, recent studies have reported that the length of the open reading frame (ORF) is altered in some genotype 1.1.2 Class I NDVs isolated at live bird markets, indicating that the virulence of Class I NDVs might have evolved and that their persistent circulation could cause the emergence of virulent NDVs that need attention [6].

**Figure 1:**
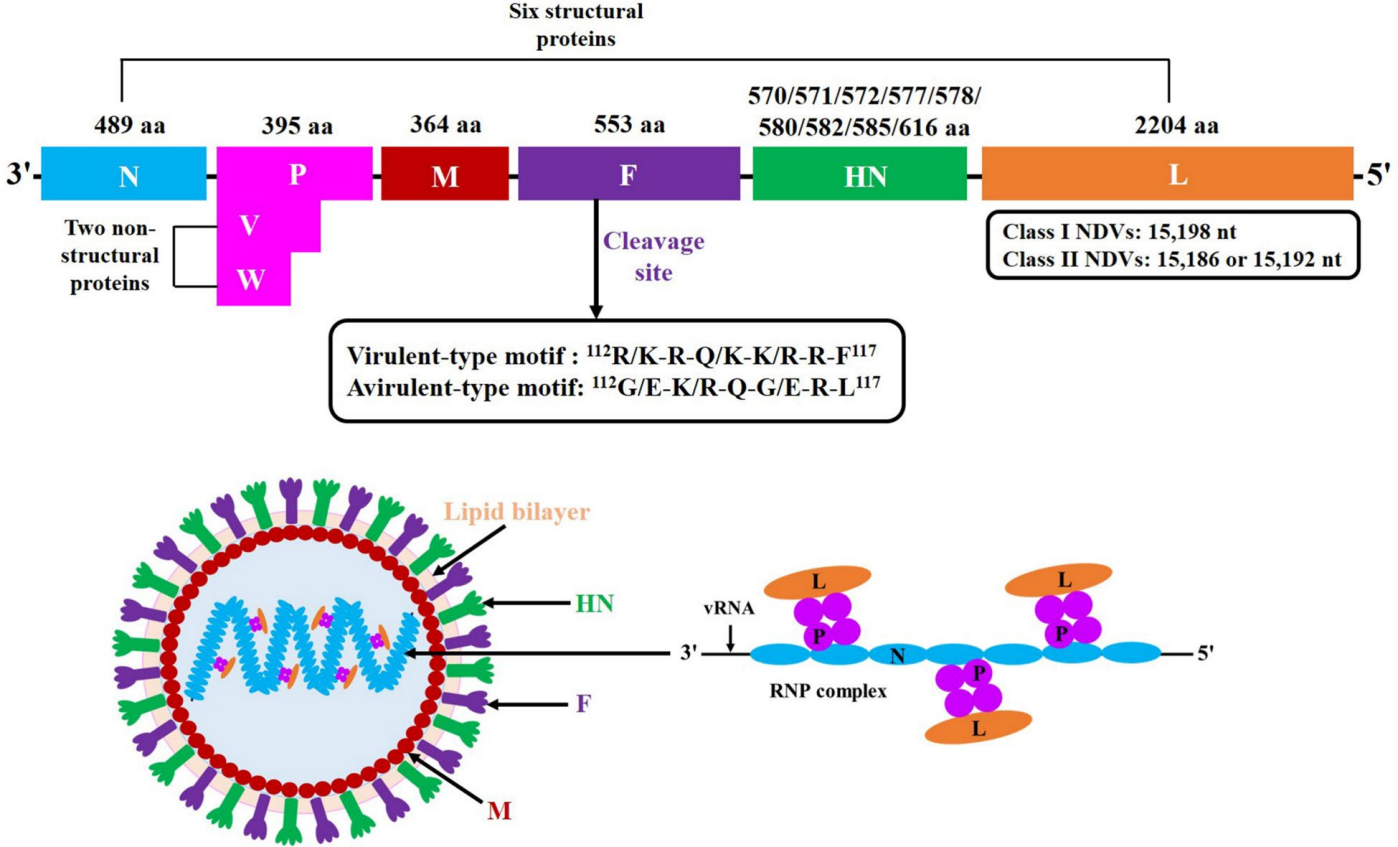
**Schematic representation of the NDV genome and virion structures.** Both Class I and II NDVs contain a nonsegmented single-stranded negative-sense genomic RNA, which comprises six major genes that encode six structural and two nonstructural proteins. The first row of numbers above the genome map indicates the aa lengths of viral proteins. The black arrow and box indicate the cleavage site of the NDV F glycoprotein with virulent- and avirulent-type motifs. The structure of NDV particles shows that the F, HN, and M proteins tightly surround the outer and inner surfaces of virions and that the N, P, and L proteins together with vRNA principally form the ribonucleoprotein (RNP) complex inside the virion.

Both Class I and Class II NDVs contain a nonsegmented single-stranded negative-sense RNA genome, which consists of six major genes in the order 3'-N-P-M-F-HN-L-5' that encode six structural proteins (known as N, P, M, F, HN, and L) and two nonstructural proteins (known as V and W) through viral *P*-gene mRNA editing [7] (Figure 1). These structural proteins constitute the major components of NDV virions, of which the glycoproteins F and HN and the nonglycosylated membrane protein M tightly surround the outer and inner surfaces of virions, and the internal N, P, and L proteins together with viral RNA (vRNA) principally form the ribonucleoprotein (RNP) complex inside the virions [7] (Figure 1). Nonstructural V and W proteins are naturally present in very small quantities in virions and are not involved in the assembly of progeny NDV virions [8, 9]. Notably, the F and HN glycoproteins are the major antigens that induce neutralizing and protective antibodies to prevent viral infection and also play multiple roles in the lifecycle of NDV, including viral adsorption, fusion and entry, intracellular replication, assembly, budding, and release [10]. Hence, studying the structure and function of these two glycoproteins is helpful for understanding the infection, replication, and pathogenicity mechanisms of NDV and developing more effective treatment strategies against NDV infection.

Current evidence has shown that other viral proteins contribute to NDV virulence in a variety of ways, but the F and HN glycoproteins justifiably serve as the major determinants of NDV virulence [11–14]. In particular, the type of F glycoprotein cleavage site is the precondition determining the virulence and pathotype of NDV, with ^112^R/K-R-Q/K-K/R-R-F^117^ for virulent NDVs and ^112^G/E-K/R-Q-G/E-R-L^117^ for avirulent NDVs [15, 16] (Figure 1). Numerous studies have reported that the F and HN glycoproteins clearly affect the tissue tropism, pathogenic phenotype, and thermostability of NDV and affect its infection, replication, and pathogenicity. Over the past 50 years, although much progress has been made in elucidating the structure and function of the NDV F and HN glycoproteins, comprehensive review studies on these proteins are lacking. Therefore, we have summarized the advancements in the understanding of the structural features and membrane fusion mechanisms of the F and HN glycoproteins, their relationships with viral virulence, pathogenic phenotype and thermostability, and the crucial roles of F/HN–host protein/compound interactions in the infection, replication, and pathogenicity of NDV. This systematic review contributes to our understanding of the replication and pathogenesis of NDV and promotes the development of antiviral strategies to efficiently prevent the infection of NDV and related paramyxoviruses.

## Structural features of the NDV F and HN glycoproteins

### Structural features of the NDV F glycoprotein

**Figure 2:**
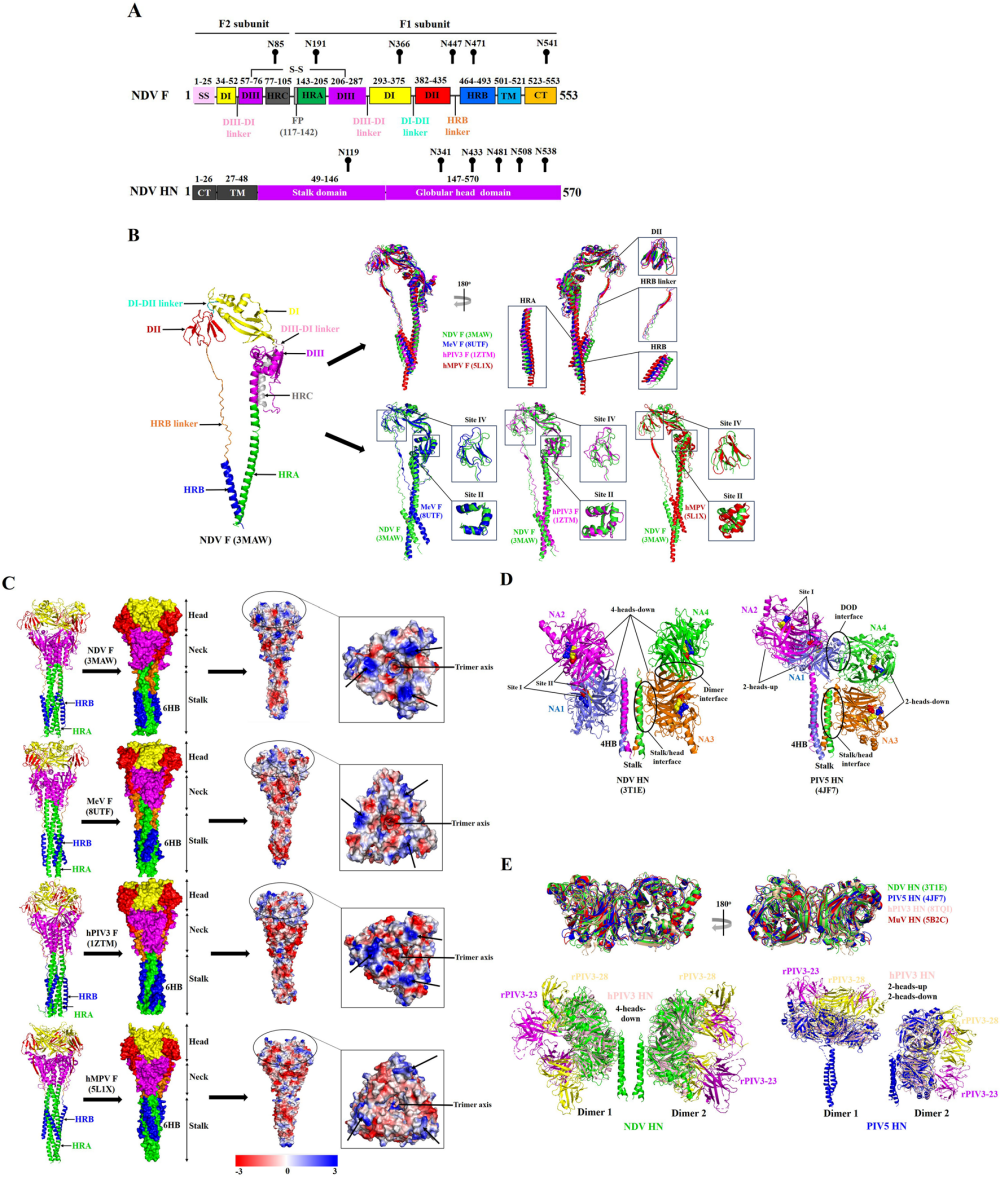
**Schematic representation of the F and HN glycoprotein structures of NDV and related paramyxoviruses. A** Schematic diagram representing the major functional domains and glycosylation sites within the NDV F and HN glycoproteins. **B** The post-fusion structure of the NDV F monomer in the cartoon drawing is coloured by domain, and the colouring scheme is identical to that in (**A**). The superimposed post-fusion structures of functional domains and antigenic sites among NDV, MeV, hPIV3, and hMPV F monomers are shown on the right. **C** Post-fusion structures and electrostatic profiles of NDV, MeV, hPIV3, and hMPV F trimers. The arrows point to the conserved regions of positive charge on the apices of the post-fusion F timers. **D** Cartoon drawing of the NDV and PIV5 HN tetramer structures in the “4-heads-down” and “2-heads-up/2-heads-down” conformations, with important sites and regions marked. **E** The superimposed globular head structures of NDV, PIV5, hPIV3, and MuV HN dimers are shown above. The superimposed structures of the hPIV3 HN (light pink) + rPIV3-23 (purple) + rPIV3-28 (yellow) complex with the “4-heads-down” NDV HN tetramer (green) or the “2-heads-up/2-heads-down” PIV5 HN tetramer (blue) are shown below. The above structure representations were generated by PyMOL 2.5.5 software.

The ORF of the *F* gene of different NDVs is 1662 nt in length and encodes a protein that is 553 aa in length with a molecular weight of approximately 68 kilodaltons (kDa). Like other paramyxoviruses, the NDV F glycoprotein is a typical type I transmembrane protein that mainly mediates viral entry into cells and viral spread by inducing virus–cell or cell–cell membrane fusion [17, 18]. In addition, the NDV F glycoprotein is a homotrimer that undergoes multiple processing processes. Briefly, each NDV F monomer is first synthesized as a biologically inactive F_0_ precursor, which is glycosylated and forms a homotrimer in the endoplasmic reticulum (ER). Next, the F_0_ homotrimers are transported through the Golgi complex and then activated by proteolytic cleavage into the disulfide-linked F1 (C-terminus)–F2 (N-terminus) complex [19, 20]. The F1 subunit contains several functional domains in the following order: the fusion peptide (FP) (aa 117–142), two hydrophobic heptapeptide repeat (HR) domains (HRA (aa 143–205) and HRB (aa 464–491)), the transmembrane (TM) domain (aa 501–521), and the cytoplasmic tail (CT) domain (aa 523–553) [21, 22] (Figure 2A). Beyond that, approximately 250 aa residues exist in the HRA and HRB domains of the F1 subunit, which can be further divided into three subdomains (known as DI, DII, and DIII) and three linkage regions known as DIII-DI, DI-DII, and HRB linkers [23] (Figure 2A). The F2 subunit contains the N-terminal cleavable signal sequence (SS) (aa 1–25), the C-terminal HRC (the third heptad repeat region) (aa 77–105), the middle DI and DIII subdomains, and the DIII–DI linker [23] (Figure 2A).

The major functions of the NDV F glycoprotein domains have been elucidated. For instance, the SS and HRC domains mainly target the F glycoprotein polypeptide to the ER and modulate its fusogenicity, respectively [24, 25]. Notably, the conserved aa residues (L81, Y84, L88, L91, L92, P94, L95, and I99) in the HRC domain are critical for normal synthesis, cell surface expression, and the monomer stability of the F glycoprotein and for the formation and conformational change of the F trimer [26]. In addition, the FP together with the HRA domains promote the refolding and fusion activity of the F glycoprotein [27, 28]. Moreover, the HRA domain can form a crimped helix in the form of a trimer when membrane fusion is triggered, and the stretched status of HRA can mediate FP insertion into the adjacent target cell membrane, thus forming a transient fusion intermediate [28]. Afterwards, the HRB combines with the HRA crimped helix in a reverse parallel direction to form a six-helix bundle (6HB), which pulls and fuses the target cell membrane and neighbouring cell membrane [18, 21, 29].

Moreover, a 21-aa TM domain and 31-aa CT domain near the C-terminus of the NDV F1 subunit are important for the structure and function of the F glycoprotein and the hyperfusogenic phenotype modulating the replication and pathogenicity of NDV, respectively [30, 31]. Notably, the tyrosine residues (Y524 and Y527) and di-leucine residues (L536 and L537) within the CT domain can regulate the cell surface expression of the F glycoprotein and modulate the budding, fusion activity, and pathogenic phenotype of NDV [32–34]. Furthermore, functional analysis of DIII–DI, DI–DII, and HRB linkers revealed that the conserved residues (I274, D277, V287, P290, and L295) around the DIII-DI linker region are critical for the folding and fusion activity of the F glycoprotein [35], and the four residues (G377, A378, L379, and T380) in the DI–DII linker region play important roles in the fusion activity of the F glycoprotein and the requirement of HN for promoting membrane fusion [36]. In addition, one recent study revealed that the four residues (L436, E439, I450, and S453) within the HRB linker can also modulate the fusion ability or cell surface expression of the F glycoprotein [37].

NDV F glycoprotein is a glycosylated protein that contains six potential N-linked glycosylation sites (N85, N191, N366, N447, N471, and N541) (Figure 2A), which are conserved in most NDVs and play vital roles in the fusogenicity of F and in the virulence, replication, and pathogenicity of NDV [38]. Previous studies have shown that mutation at each of the five glycosylation sites (N85, N191, N366, N447, and N471) does not affect the cleavage or cell surface expression of the F glycoprotein [39]. Moreover, mutation at residues N85, N191, N366, and N471 does not affect viral syncytium formation, but recombinant NDVs harbouring these mutations are obviously attenuated; conversely, mutation at residue N447 increases viral virulence and syncytium formation [39]. However, the mutant virus bearing the mutant N541 glycosylation site could not be successfully rescued, suggesting its importance in viral viability. In addition, the double mutation at residues N191 and N471 results in increased viral virulence, fusogenicity, and replication efficiency, indicating their negative regulatory effects on the replication and pathogenicity of NDV [39].

NDV F glycoprotein also contains 13 cysteine (C) residues (C27, C76, C199, C338, C347, C362, C370, C394, C399, C401, C424, C514, and C523), which participate in disulfide bond formation in the F glycoprotein [40, 41]. In addition to the three variational C residues (C27, C514, and C523), the other 10 C residues are highly conserved among NDV and other paramyxoviruses, suggesting that folding of the molecule and intramolecular disulfide bonds are crucial to the structure and function of the paramyxovirus F glycoproteins [18, 42, 43].

The F glycoprotein of paramyxoviruses (including NDV) undergoes a large, irreversible conformational change/refolding event from the prefusion form to the post-fusion form, promoting virus‒cell membrane fusion and opening a pore to deliver the viral genome into the cytoplasm. To date, the post-fusion F conformation of most paramyxoviruses (represented by NDV, measles virus (MeV), human parainfluenza virus 3 (hPIV3), and human metapneumovirus (hMPV)) has been solved, but the prefusion form of these viral F glycoproteins is still lacking. Analysis of the global superposition of the NDV, MeV, hPIV3, and hMPV post-fusion F monomers revealed that there is generally good correspondence among their crystal structures (Figure 2B). However, partial differences in these viral F structures were still observed, as the greatest differences in the best global alignment were located in the DII and HRB regions, and deviations in the HRA and HRB linker regions were also evident (Figure 2B). Nonetheless, these discrepant regions are closely related to the conformational transitions and important for the membrane fusion of each viral F glycoprotein [23, 44–46].

In addition, studies of the F glycoproteins of respiratory syncytial virus (RSV) and hMPV have suggested that there are six major antigenic sites known as sites Ø, I, II, III, IV, and V on the viral F glycoprotein, of which sites Ø and V are found only in the prefusion conformation, whereas sites II and IV are similar between the prefusion and post-fusion conformations [47]. Notably, antigenic sites II and IV are highly similar and well conserved between the RSV and hMPV F glycoproteins because of the structural similarity of their overall post-fusion structures [45]. However, in addition to the conserved site II between NDV and MeV, sites II and IV of the NDV F glycoprotein clearly differ from those of the MeV, hPIV3, and hMPV F glycoproteins (Figure 2B). The inconsistent structures of sites II and IV between NDV and other paramyxovirus members indicate that a single antibody against either of the sites is less likely to neutralize all paramyxovirus members [47].

The soluble post-fusion trimeric structures of the NDV, MeV, hPIV3, and hMPV F glycoproteins have been resolved. The results revealed that these viral F trimers are formed by three narrow and elongated monomers, which make up a globular head region that is predominantly a β-sheet, a neck region formed by both α-helices and a β-sheet, and a stalk region principally containing α-helices (Figure 2C). Moreover, the viral F trimer structures contain highly stable and characteristic 6HB formed by the HRA and HRB regions at the base of the stalk, and there is also a common negatively charged surface in their stalk regions (Figure 2C). In addition, the head regions of the NDV, MeV, hPIV3, and hMPV F trimers are formed by lateral interactions between the DI and DII domains, and the top view of their F heads reveals a triangular shape and a central cavity that is negatively charged with three positively charged lobes projecting from the trimer axis (Figure 2C). Taken together, these findings suggest that the post-fusion conformation of paramyxovirus F glycoproteins has a similar structure and function, but whether these partial structural variations and electrostatic profiles affect membrane fusion progression remains to be further explored.

### Structural features of the NDV HN glycoprotein

The ORF of the *HN* gene varies in length from 1713 to 1851 nt in different NDVs and encodes at least nine different length variants, including 570 aa, 571 aa, 572 aa, 577 aa, 578 aa, 580 aa, 582 aa, 585 aa, and 616 aa variants (Figure 1). The length diversity of the HN glycoprotein arises from differences in the position of the stop codon of the NDV *HN* gene ORF, and each length can represent a viral lineage and virulence pathotype [48]. The HN with 577 aa can be found in moderately virulent and avirulent NDVs, whereas the HN with 571 aa or 616 aa is present only in highly virulent and avirulent NDVs. However, only the HN glycoprotein with 616 aa (the HN_0_ precursor) requires post-translational cleavage of the C-terminal 45 aa for its activation [49]. In addition, studies have shown that the replication and pathogenicity of a lentogenic NDV Clone30 were not affected by length variations in the 571 aa, 577 aa, and 616 aa variants of the HN glycoprotein [50], but the C-terminus 39-aa truncation of HN increased the virulence and immunogenicity of another lentogenic NDV V4 [51]. Moreover, several other studies have reported that the C-terminal extension of the HN glycoprotein does not alter viral tissue tropism but may contribute to reduced virulence in some lentogenic NDVs [52, 53].

In contrast, another study revealed that different lengths of the HN glycoprotein do not affect the virulence of recombinant virulent NDVs, whereas the C-terminal extension of the HN glycoprotein increases viral haemagglutination titre and receptor binding ability but impairs viral neuraminidase (NA) activity, fusogenic activity, and replication ability [54]. This study also demonstrated that recombinant NDVs bearing the 566 aa or 567 aa HN glycoprotein could be successfully rescued, but a recombinant virus harbouring the 565 aa HN glycoprotein could not be recovered. Moreover, a sequence revertant of the 566 aa HN glycoprotein was recovered to the original 570 aa after serial passaging in embryonated chicken eggs [54]. These results indicate that shorter HN glycoproteins occur evolutionarily from a longer ancestral HN because of the introduction of translational stop codons and that the length of the HN cannot be reduced indefinitely, thereby demonstrating that the natural length of the HN glycoprotein is evolutionarily conserved and optimal for the corresponding NDVs [54].

The HN glycoprotein of NDV and other paramyxoviruses (represented by PIV5 and hPIV3) is a typical II homotetrameric transmembrane protein that contains both haemagglutinin (HA) and NA activities and plays essential roles in viral attachment, virus–cell membrane fusion, and viral release and dissemination [10, 40]. Like the PIV5 and hPIV3 HN glycoproteins [55, 56], the NDV HN glycoprotein is composed of four major domains, including the N-terminal CT (aa 1–26) and TM (aa 27–48) domains, followed by a stalk domain (aa 49–146) and a C-terminal globular head domain (aa 147–570) [57] (Figure 2A). Functional analysis revealed that the CT domain of HN contains 26 highly conserved residues, which participate in the proper membrane insertion of the HN glycoprotein and its interaction with the M protein [58, 59]. However, the first 2-aa in the CT domain can be deleted without affecting viral biological characteristics, but the deletion of the first 4 aa or mutation of the sixth residue (S6A) obviously reduces the colocalization of HN and M proteins and attenuates the fusogenicity and pathogenicity of NDV, suggesting that these residues within the CT domain are important for virion incorporation, cell fusion, and pathogenicity [60]. The adjacent TM domain principally maintains the tetrameric structure of the HN glycoprotein, and residue mutations in the TM domain in particular diminish the attachment and fusion-promoting activities of the NDV HN glycoprotein [61].

The stalk domain predominantly affects the fusion-promoting activity of the HN protein and mediates the interaction of HN with the homotypic F glycoprotein. In addition, the HN glycoprotein harbouring L94A, L96A, and L97A mutations in the stalk domain still possesses NA activity and full attachment activity but no fusion-promoting activity, indicating that the fusion-promoting activity of the HN glycoprotein does not correlate with the level of its NA activity [62]. As the largest and most dominant functional region of the NDV HN glycoprotein, the globular head domain is connected to the stalk domain through a short flexible unstructured linker that allows the globular head to take different positions to facilitate F–HN interaction and F-triggering membrane fusion [63, 64] (Figure 2D). Several studies have shown that the globular head is involved mainly in receptor recognition and NA activity [65, 66]. In addition, the globular head contains two sialic acid receptor binding sites known as “site I” and “site II” [67], of which site I is correlated with receptor binding (the key residues are E400, R415, and Y525) and NA activity [68], and site II is relevant for receptor-binding and fusion-promoting activities (the key residues are C172, R174, C196, D198, Y526 and E547) [69–71]. Notably, residue Y526 is crucial for the functional activity of the HN glycoprotein and the replication and pathogenicity of NDV, as the Y526Q mutation significantly reduces receptor binding, fusion and NA activity and attenuates viral virulence, replication efficiency, and pathogenicity [72].

Like the viral F glycoprotein, the HN glycoprotein of NDV and other paramyxoviruses is also a glycosylated protein, and glycosylation is important for its proper folding, stability, intracellular transport, and receptor binding [73, 74]. The NDV HN glycoprotein contains six potential N-linked glycosylation sites (N119, N341, N433, N481, N508, and N538) distributed in the stalk and globular head domains [75] (Figure 2A). Apart from the N508 site, which is absent in some NDVs, the other five glycosylation sites are relatively conserved among different NDVs. Moreover, except for site N538, which is inaccessible for glycosylation, the four sites N119, N341, N433, and N481 are generally used in NDVs [73, 76]. The results of site-specific heterogeneity of glycan composition analysis revealed that two main classes of oligosaccharides, complex-type and high-mannose-type glycans, exist in the NDV HN glycoprotein [77]. Importantly, the removal of N-glycans from the HN glycoprotein clearly affects its exocytic transport and surface expression and the pathogenicity of NDV [73, 76], whereas the addition of N-glycans in the stalk domain of the HN glycoprotein blocks its interaction with the F glycoprotein and prevents F-triggering fusion [78].

Beyond that, conserved O-linked glycosylation sites, T71, T62, and T88, have been identified in the HN stalk domain of NDV, PIV5, and hPIV1, respectively [57, 77], which may influence the oligomerization of HN or the interaction between HN and F and affect viral fusion kinetics. In addition, the NDV HN glycoprotein also contains 14 C residues (C123, C172, C186, C196, C238, C247, C251, C344, C455, C461, C465, C531, C542, and C596). These C residues are well conserved among NDVs and are important for the maturation of the HN glycoprotein, with the exception of residue C123 [79]. Notably, residue C123 is involved in disulfide-linked dimer formation, but the HN glycoprotein lacking C123 still forms a dimer and tetramer and can be efficiently transported to the cell surface [80]. However, a recombinant NDV harbouring the mutant HN (W123C) together with a mutant F glycoprotein (C27R) exhibits increased virulence and pathogenicity, demonstrating that these mutations are more beneficial for the formation of disulfide-linked HN dimers and the pathogenic phenotype of NDV [81].

The HN glycoproteins of NDV, PIV5, hPIV1–4, mumps virus (MuV), and Sendai virus (SeV) have been shown to form active tetramers, whereas its NA domain primarily exists as a monomer [43, 67]. To date, only the HN glycoproteins of NDV and PIV5 have a complete tetrameric structure for both the head and the stalk regions. Analysis of their crystal structures revealed that the globular head domain is composed of four six-bladed β-sheet propeller fold monomers (NA1, NA2, NA3, and NA4), and each monomer contains a centrally located site I that also has NA activity [55, 57] (Figure 2D). In addition, site II was found to be located at the dimer interface of the NA domains in the globular head of NDV HN rather than PIV 5 HN, which is composed of hydrophobic residues from both monomers and interacts with sialic acid but lacks NA activity (Figure 2D).

In addition, the crystal structures also reveal the tetramerization of the NDV and PIV5 HN stalks, known as the four-helix bundle (4HB), in a helical coiled-coil form (Figure 2D), which consists of a hydrophobic core formed by an 11-residue repeat [55, 57]. However, the 4HB stalk of NDV HN glycoprotein is packed between its two contactless globular head dimers, and the dimeric heads are backfolded onto the stalk, with the lower head of each dimer making short-range contact with the upper part of the stalk domain, thus constituting a “4-heads-down” conformation (Figure 2D) [57]. In contrast, there are two disulfide-linked heads in the upper position and two heads in the lower position, which adopt a “2-heads-up/2-heads-down” conformation in the PIV5 HN glycoprotein. Correspondingly, this structure exhibits a novel noncovalent dimer-of-dimers (DOD) interface between one up head and one down head [55] (Figure 2D). Moreover, a similarly sized stalk/head interface is observed in the structure of the NDV and PIV5 HN glycoproteins (Figure 2D), but the sequence alignment of NDV with PIV5 and hPIV1-4 HN stalks indicates that the F-activation residues are only partially conserved and may also contribute to HN–F-specific interactions [57].

Comparison analysis of the NDV, PIV5, hPIV3, and MuV HN dimeric globular heads revealed that their crystal structures generally correspond well [55, 57, 82, 83] (Figure 2E). Moreover, accommodation of the four Fabs (two rPIV3-23 Fabs and two rPIV3-28 Fabs) bound to the hPIV3 HN dimer does not cause substantial rearrangements, which also appears compatible with the tetrameric “4-heads-down” and “2-heads-up/2-heads-down” HN conformations observed for the related NDV and PIV5 HN glycoproteins, respectively (Figure 2E). Notably, the two conformations are also appropriate for the newly solved crystal structures of other paramyxovirus attachment proteins, including the Nipah virus (NiV) G and canine distemper virus (CDV) H glycoproteins [84, 85], although certain differences are present in the mechanism of HN/G/H head-mediated receptor engagement to activate F glycoprotein in some paramyxoviruses [86–88]. Nonetheless, these findings provide novel insights into the development of broad-spectrum antibodies or antiviral drugs targeting the HN glycoprotein to block paramyxovirus infection.

## Membrane fusion mechanism of the NDV F and HN glycoproteins

Viral entry into host cells is a necessary step for paramyxoviruses to carry out their lifecycle. The membrane fusion efficiency of the triggering of the paramyxovirus (including NDV, PIV5, hPIV1–4, and MuV) F glycoprotein by the HN glycoprotein critically affects the degree of viral entry [89]. The consensus is that virus-induced membrane fusion requires the active participation of both the HN and F glycoproteins for most paramyxoviruses, with the exceptions of hMPV, NiV, and Hendra virus (HeV). It has been shown that the paramyxovirus HN glycoproteins need to stabilize the F glycoprotein to prevent viral inactivation before the cell receptor is engaged, and then the stalk domain of the viral HN glycoprotein communicates with site I and/or II in its globular head to activate the F trimer once the host cell receptors have been engaged [43]. Afterwards, the trimeric F glycoprotein undergoes further dramatic structural rearrangements from the prefusion state to the post-fusion conformational state to drive virus–cell membrane fusion [86, 87, 89]. Thus, the membrane fusion and entry of most paramyxoviruses are determined through a series of cooperative steps mediated by both the F and HN glycoproteins.

**Figure 3:**
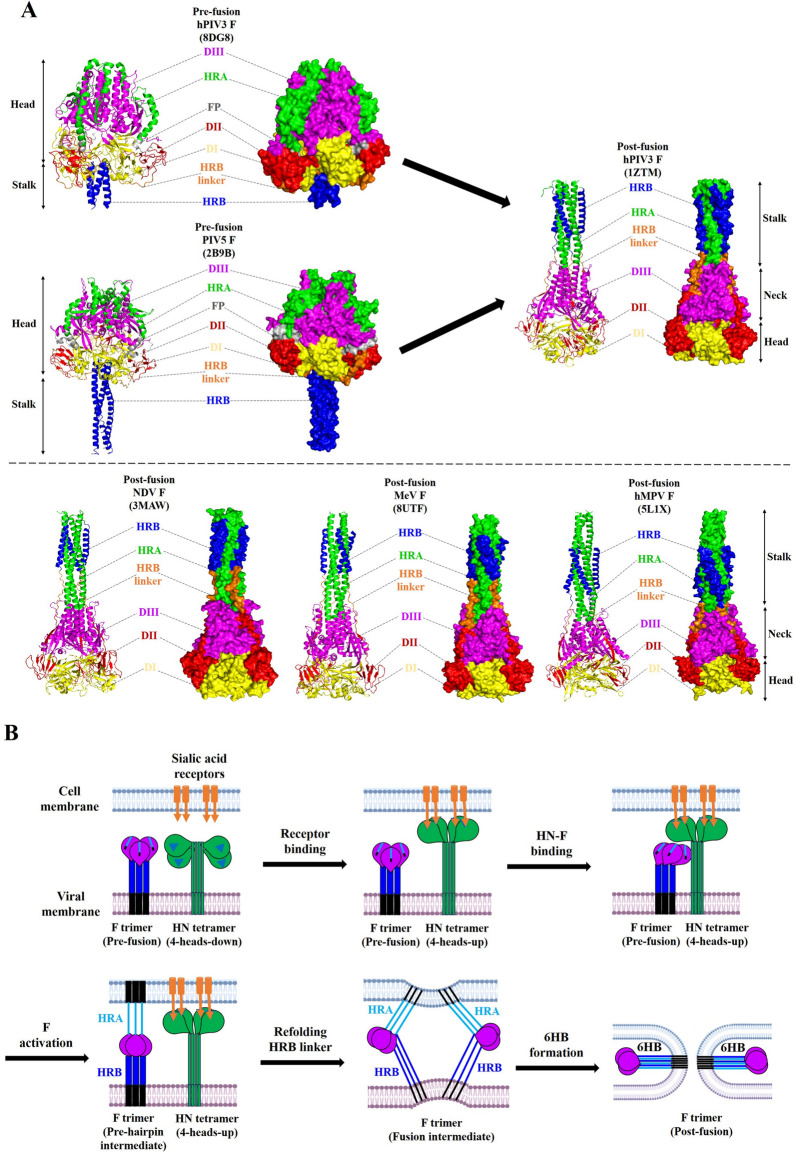
**Schematic representation of membrane fusion mediated by the NDV F and HN glycoproteins.** **A** Structural difference between the prefusion states of hPIV3 and PIV5 F trimers and the post-fusion states of hPIV3, NDV, MeV, and hMPV F trimers. The major functional domains within the hPIV3, PIV5, NDV, MeV, and hMPV F trimers are colour coded as indicated in Figure 2A. The structure representations were generated by PyMOL 2.5.5 software. **B** Schematic diagram depicting the membrane fusion mechanism mediated by the NDV F and HN glycoproteins. NDV HN glycoprotein interacts with F glycoprotein after it binds to cell surface receptors, which then causes the rearrangement of the prefusion F glycoprotein and leads to FP insertion into the cell membrane. After that, the prefusion F glycoprotein refolds into a post-fusion conformation through a series of fusion intermediates, ultimately driving viral–cellular membrane fusion.

Like other paramyxovirus F glycoproteins, the NDV F glycoprotein also contains two different conformational states in virus-infected cells, of which the prefusion F trimer is a metastable and high-energy conformation formed by the newly synthesized F monomers folded in the ER, and the post-fusion F trimer is a highly stable and low-energy conformation when the HN glycoprotein binds to the cell surface receptor followed by an ATP-independent F conformation change [23, 25]. Therefore, the conversion of high energy to low energy is believed to drive the membrane fusion process during F trimer refolding [90]. The crystal structures of most paramyxovirus post-fusion trimeric F proteins have been determined, but prefusion F trimers are generally not available. However, comparisons of prefusion F structures from other paramyxoviruses, such as hPIV3, PIV5, HeV, and Langya virus (LayV), indicate that the prefusion F trimers are highly similar among paramyxoviruses [44, 91–94]. With the prefusion F trimer of hPIV3 and PIV5 as the prototype, the crystal structure of the prefusion F state appears to have a “tree”-like fold, which is characterized by a large globular head composed of four main domains (DI, DII, DIII, and HRA) per subunit and a short stalk region composed of three HRB domains assembled in a 3HB structure [44, 95] (Figure 3A). The 11 distinct HRA segments (four α-helices, two β-sheets, and five loops) as well as the DIII domain are predominantly located at the top of the globular head, whereas the DI and DII domains mainly constitute the base of the head (Figure 3A). Moreover, the hydrophobic FP region is mostly buried in the prefusion structure and clamped by the DII and DIII domains of two different monomers within the globular head region [91] (Figure 3A).

In addition, comparison of the prefusion (hPIV3 and PIV5) and post-fusion (hPIV3, NDV, MeV, and hMPV) F trimers revealed obvious structural rearrangements of these domains, revealing that the whole DI and most DII domains form the top of the globular head, that the entire DIII and partial DII domains are located mainly in the neck region, and that the 11 HRA segments are converted from the head to the stalk region to form the extended α-helical 6HB fold together with the 3HB structure [96] (Figure 3A). Notably, from the current structures of paramyxovirus prefusion and post-fusion F trimers, the FP region can be simply located at the N-terminus of the HRA coiled-coil structure and concomitantly inserted into the anchoring membrane with the TM domain at the time of F_0_ cleavage, but it is invisible in the post-fusion state (Figure 3A). According to the conformational changes of the PIV5 F glycoprotein and influenza virus HA protein in the transition from the pre- to post-fusion state, it can be concluded that the FP away from the viral membrane and TM domain is conducive to making the HRA and HRB coiled-coils form a stable 6HB structure [95, 97]. However, the precise trigger of FP that promotes metastable F prefusion in its transition to the 6HB and post-fusion structure remains to be explored.

It is accepted that the paramyxovirus HN glycoproteins undergo structural conformational changes as a consequence of cell receptor engagement, which eventually leads to the activation of the F glycoprotein at the right time and place. Currently, five main models, including the sliding model, the stalk-exposure/induced fit model, the safety-catch model, the bidentate attachment protein/F interaction model, and the receptor-induced oligomerization model, have been proposed to elucidate how HN tetramers move to actively trigger F trimers [90, 96]. Among these, the stalk-exposure/induced fit model for NDV and PIV5 highlights the receptor-dependent constitutive bioactivity mediated by head attachment protein constructs, which may represent the shared membrane fusion and viral entry mechanism of paramyxoviruses. In this model, the HN tetramer presents the “4-heads-down” conformation, where two lower heads of each dimeric head unit make physical contact with the C-terminal region of the HN stalks, thus directly covering the F-binding/activation sites and preventing intracellular HN/F interaction [57] (Figure 3B). Upon binding to sialic acid receptors at the cell surface, the HN tetramer undergoes a conformational change from the inactive “4-heads-down” state to the activated “4-heads-up” state, which exposes the F-activating region within the HN stalk for physical interaction with the F glycoprotein [98] (Figure 3B). After F glycoprotein activation is triggered, the HRA adjacent to the FP undergoes a major conformational change that extends the FP outwards for insertion into the target cell membrane. This process leads to the formation of a prehairpin intermediate, which simultaneously anchors the F glycoprotein to the viral and cell membranes [99] (Figure 3B).

After the formation of the fusion intermediate caused by HRB linker refolding, the HRB adjacent to the TM domain translocates to bind the HRA in an antiparallel pattern, thereby forming a stable 6HB that draws the two membranes close enough to undergo membrane fusion [100] (Figure 3B). Additionally, although the stalk-exposure/induced fit model for NDV F activation and HN/F-mediated membrane fusion may present features common to those of all paramyxovirus members, many lines of evidence indicate that the membrane fusion machinery of hPIV3, MeV, CDV, NiV, and HeV can already form intracellularly [84, 100–102]. Therefore, additional structural information, including the HN tetramer in complex with cell receptors, the uncleaved and cleaved metastable F trimer, the pre- and post-fusion F trimer from the same virus species, the biochemical changes in HN/F interaction as fusion proceeds, and any F trimer in complex with HN-stalk or the stalk domain itself, may be needed to verify the differences in receptor engagement and F activation, as well as the events that promote membrane fusion among different paramyxoviruses.

## Virulence, pathogenic phenotype, and thermostability correlations of the NDV F and HN glycoproteins

### The F glycoprotein cleavage site is a prerequisite for NDV virulence

Previous studies have shown that different NDV pathotypes are associated with the motifs of the F glycoprotein cleavage site (Fcs), as cleavage of the F_0_ precursor protein harbouring the furin motif into the F1 and F2 subunits by host proteases is essential for progeny NDVs to become infective and efficiently replicate in host cells and tissues [49, 103, 104]. It was found that the virulent (velogenic and mesogenic) NDVs have a polybasic aa motif (^112^R/K-R-Q/K-K/R-R-F^117^) in the Fcs that can be cleaved intracellularly by ubiquitous furin-like proteases, which confers NDVs with the ability to replicate in a wide variety of tissues, whereas the avirulent (lentogenic) NDVs have a monobasic aa motif (^112^G/E-K/R-Q-G/E-L^117^) in the Fcs that can be only cleaved extracellularly by trypsin-like proteases, which restricts viral replication to the respiratory and enteric tracts. Consequently, the Fcs motifs are considered to be the primary determinant for NDV virulence and differentiate the virulent NDVs from the avirulent NDVs, but the association of Fcs motifs with NDV virulence remains unclear.

Since the establishment of NDV reverse genetics manipulation in 1999, the role of Fcs motifs in the virulence and pathotype of NDV has been extensively investigated (Table 1). The first evidence is that a genetically tagged derivative of lentogenic NDV LaSota (rNDFLtag), in which the original Fcs motif (GRQGRL) was changed to the consensus cleavage site of virulent NDVs (RRQRRF), has dramatically increased virulence (the intracerebral pathogenicity index (ICPI) value increased from 0.00 to 1.28) [15]. Later, several other research groups demonstrated that substitution of the Fcs motifs (G/E-R-Q-G/E-R-L) of avirulent NDVs with the Fcs motifs (R/K-R-Q/K-K/R-F) of virulent NDVs significantly increases the virulence (represented by increased ICPI, intravenous pathogenicity index (IVPI) values, and/or decreased mean death time (MDT) value) and pathogenicity of recombinant NDVs and changes the viral pathogenic phenotypes [49, 105–109]. Conversely, recombinant mesogenic and velogenic NDVs harbouring the Fcs motifs of lentogenic NDVs exhibit decreased virulence and pathogenicity and altered pathogenic phenotypes [110–117]. In addition, researchers have replaced the Fcs motif of velogenic NDV rBan/010 with the Fcs motifs of avirulent AMPV-2, AMPV-7, and APMV-8 to generate several recombinant NDVs (rBan-APMV2, rBan-APMV7, and rBan-APMV8), but the viral ICPI values were clearly lower than those of the parental virus, which satisfies the standard of lentogenic NDVs [118]. Therefore, these results undoubtedly confirm the previous assumption that the Fcs motif is an important determinant of NDV virulence.

**Table 1:** **Properties of recombinant NDVs harbouring aa mutations in the Fcs motif**

Virus	Parent	Pathotype^†^	Fcs motif	MDT^‡^	ICPI	IVPI^‡^	Reference
rNDFL	LaSota	Lentogenic	GRQGRL	nd	0.00	nd	[15]
rNDFLtag^*^		Mesogenic	RRQRRF	nd	1.28	nd	
rNDV	Clone-30	Lentogenic	GRQGRL	nd	0.01	nd	[49]
rNDVF1^*^		Mesogenic	RRQKRF	nd	1.28	nd	
rLaSota	LaSota	Lentogenic	GRQGRL	nd	0.00	0.00	[105]
rLaSota V.F.^*^		Mesogenic	RRQKRF	nd	1.12	0.00	
Alaska/415	Alaska/415/91	Lentogenic	ERQERL	> 120 h	0.00	0.00	[106, 107]
rNDV/9a5b-D5C1^*^		Velogenic	KRQKRF	54 h	1.89	2.72	
rNDV F0	LaSota	Lentogenic	GRQGRL	107 h	0.01	nd	[108]
rNDV F3aa^*^		Mesogenic	RRQRRF	52 h	1.42	nd	
rNDV F3aa-S		Mesogenic	RRQRRS	58 h	1.27	nd	
rNDV	Clone-30	Lentogenic	GRQGRL	nd	0.01	nd	[109]
rNDV (Fcs)^R75/98*^		Mesogenic	RRKKRF	nd	1.36	nd	
rNDV/ZJ1	ZJ1	Velogenic	RRQKRF	54 h	1.89	2.70	[110]
rNDV/ZJ1FM^**^		Lentogenic	GRQERL	> 120 h	0.13	0.00	
rNDV/I4	I4	Velogenic	RRQKRF	48 h	1.96	2.55	[111]
rNDV/AI4^**^		Lentogenic	GRQGRL	> 120 h	0.13	0.00	
rSG10	SG10	Velogenic	RRQKRF	45 h	1.89	nd	[112]
raSG10^**^		Lentogenic	GRQGRL	> 120 h	0.25	nd	
rNA-1	NA-1	Velogenic	RRQKRF	59 h	1.90	nd	[113]
rmNA-1^**^		Lentogenic	GRQGRL	132 h	0.00	nd	
rG7	G7	Velogenic	RRQKRF	44 h	1.60	2.40	[114]
rG7-E1954Q + F^**^		Lentogenic	GRQGRL	> 120 h	0.20	0.00	
rSD19	SD19	Velogenic	RRQKRF	58 h	1.70	nd	[115]
raSD19^**^		Lentogenic	GRQGRL	> 120 h	0.16	nd	
rHR09	HR09	Velogenic	RRQRRF	58 h	1.80	nd	[116]
rcHR09-CI^**^		Lentogenic	ERQERL	> 120 h	0.00	nd	
rcHR09-CII^**^		Lentogenic	GRQGRL	> 120 h	0.00	nd	
rBan/010	Ban/010	Velogenic	RRQKRF	51 h	1.88	nd	[117, 118]
rBan/AF^**^		Lentogenic	GRQGRL	> 120 h	0.00	nd	
rBan-APMV2		Lentogenic	KPASRF	nd	0.00	nd	
rBan-APMV7		Lentogenic	LPSSRF	nd	0.25	nd	
rBan-APMV8		Lentogenic	YPQTRL	nd	0.00	nd	
rNDFLtag	LaSota	Mesogenic	RRQRRF	nd	1.30	nd	[16]
rNDFLtag-F^FM^		Lentogenic	RRQRRL	nd	0.50	nd	
rNDFLtag-F^FM2^		Lentogenic	RRQGRF	nd	0.30	nd	
rNDFLtag-F^FM3^		Mesogenic	RGQRRF	nd	1.10	nd	
rNDFLtag-F^FM4^		Lentogenic	RKQKRF	nd	0.50	nd	
rNDV	Beaudette C	Velogenic	RRQKRF-I	nd	1.58	nd	[121]
rNDV-Q114R		Mesogenic	RRRKRF-I	nd	1.33	nd	
rNDV-Q114R/I118V		Mesogenic	RRRKRF-V	nd	1.33	nd	
rNDV-Q114R/K115R		Mesogenic	RRRRRF-I	nd	1.36	nd	
rNDV-Q114R/K115R/I118V		Mesogenic	RRRRRF-V	nd	1.37	nd	

^*^The lentogenic NDV Fcs motif was artificially modified into the velogenic NDV Fcs motif. ^**^The velogenic NDV Fcs motif was artificially modified into a lentogenic NDV Fcs motif. ^†^Recombinant NDVs with ICPI values > 1.60 are defined as velogenic, those with ICPI values of 0.60–1.50 are mesogenic, and those with ICPI values < 0.50 are lentogenic. ^‡^nd indicates that MDT and IVPI were not determined.

Because the Fcs motif of virulent NDVs has diverse aa sequence compositions, the individual aa site at the Fcs motif has also been assessed for its role in NDV virulence. The results revealed that recombinant NDVs rNDFLtag-F^FM^, rNDFLtag-F^FM2^, rNDFLtag-F^FM3^, and rNDFLtag-F^FM4^ harbouring the mutational residues F117L, R115G, R113G, or R113K/R115K all presented decreased ICPI values (Table 1) and exhibited delayed morbidity and mortality in comparison to the parental virus rNDFLtag [16]. Notably, these mutant NDVs clearly increased virulence after one passage in chicken brains, and their Fcs motifs reverted to the virulent-type motif (RRQRRF), with the exception of the double mutant of rNDFL-F^FM4^ (RRQKRF), suggesting that residues R113, R115, and F117 in the Fcs motif are required for the virulence and pathogenicity of NDV [16]. In addition, another study generated the F117S mutation in the Fcs motif of mesogenic rNDV F3aa and reported that the ICPI value of the mutant virus rNDV F3aa-S decreased from 1.42 to 1.27 [108], further demonstrating the important role of residue F117 in NDV virulence.

Notably, residue Q114 is present at the Fcs motif of most virulent and avirulent NDVs, except for some NDVs isolated from apparently healthy unvaccinated chickens in Africa (^112^RRRKRF-I/V^118^) and Madagascar (^112^RRRRRF-I/V^118^) [119, 120]. Moreover, some of these NDVs also contain valine (V) at position 118 of the adjacent Fcs motif instead of isoleucine (I) present in most virulent and avirulent NDVs, which is not consistent with the previous assumption that the number of basic aa residues at the Fcs motif determines the virulence of NDV [119, 120]. Thus, the role of Q114 and/or K115 and I118 in the virulence, replication, and pathogenicity of NDV was systematically evaluated in a subsequent study. The ICPI values of the recombinant NDVs rNDV-Q114R, rNDV-Q114R/I118V, rNDV-Q114R/K115R, and rNDV-Q114R/K115R/I118V were significantly lower than those of the parental virus rNDV (Table 1), and the replication capacity, pathogenicity, and cleavage processing of the F_0_ protein of recombinant NDVs harbouring Q114R and/or I118V mutations were greatly weakened, which demonstrated that residue Q114 in conjunction with residue I118 plays essential roles in the virulence and pathogenicity of NDV by affecting efficient proteolytic processing of the F_0_ protein [121]. Consequently, these results indicate that the natural Fcs motifs in the corresponding NDVs may represent the optimal aa sequence for the efficient cleavage of the F_0_ precursor protein by host proteases and virulence, replication, and pathogenicity of NDV itself [122].

However, several studies have also demonstrated that the Fcs motifs are not always consistent with the virulence and pathogenicity of NDV. For example, evidence has shown that individual NDVs have lentogenic Fcs motifs but are highly pathogenic in chickens [123], and most pigeon-origin NDVs with velogenic Fcs motifs have low ICPI values and cause only mild or no disease in chickens [124, 125]. Moreover, compared with the parental virus LaSota or rNDFL, the recombinant mesogenic NDV rNDFLtag (generated from the lentogenic LaSota) with a virulent Fcs motif exhibited only slightly increased disease severity and viral distribution [126]. These results suggest that the virulence and pathogenic pathotype of NDVs are not solely dependent on the Fcs motifs and that other viral factors may contribute to viral virulence and pathogenicity. Accordingly, researchers have further explored the different regions of the NDV F glycoprotein that possibly modulate viral pathogenicity.

A previous study revealed that a recombinant lentogenic NDV rNDV (ICPI value of 0.01) harbouring the full-length F glycoprotein from a mesogenic pigeon-origin NDV R75/98 (ICPI value of 1.10) has a lentogenic pathotype with an ICPI value of 0.60 [109]. In contrast, replacement of different F regions of rNDV with those of R75/98 revealed that rNDV(F_112-553_)^R75/98^ can be recognized as a lentogenic pathotype with ICPI values of 0.59, whereas rNDV(Fcs)^R75/98^, rNDV(F_1-117_)^R75/98^, rNDV(F_1-527_)^R75/98^, and rNDV(F_528-553_)^R75/98^ exhibit ICPI values of 1.36, 1.13, 1.36, and 1.13, respectively, which are higher than those of the parental virus R75/98 but still representative of mesogenic pathotypes [109]. Importantly, compared with the parental viruses, most of the chimeric NDVs exhibit markedly decreased replication efficiency and syncytia formation, indicating the importance of different F regions in modulating NDV virulence and pathogenicity [109]. In addition, another study revealed that two residues, D479 and S486, within the HRB domain of the virulent NDV F glycoprotein together with the unique Fcs motif (RRQRRL) are critical for increasing viral virulence and fusogenic activity [127]. Together, these findings demonstrate that the Fcs motifs determine whether an NDV is a virulent or avirulent pathotype and serve as a molecular basis and prerequisite for NDV virulence, but other regions or residues of the F glycoprotein also influence NDV virulence [128, 129].

### F and HN glycoproteins codetermine NDV virulence and pathogenic phenotype

As mentioned above, the Fcs motifs do not completely determine the virulence and pathogenic pathotype of NDV; thus, chimeric viruses bearing an *HN* and/or *F* gene exchange between different NDVs were subsequently evaluated (Table 2). The results of *HN* gene exchange between velogenic rBC and lentogenic rLaSota revealed that the chimeric virus rBC(HN)^LaSota^ harbouring the LaSota *HN* gene is markedly less virulent than the parental virus rBC is, whereas the reciprocal chimeric virus rLaSota(HN)^BC^ is moderately virulent compared with its parental virus rLaSota [130] (Table 2). Moreover, compared with those inoculated with rBC and rLaSota, chickens infected with rBC(HN)^LaSota^ and rLaSota(HN)^BC^ have much higher and lower survival rates, respectively [130]. More specifically, the viruses rBC and rLaSota(HN)^BC^ are widely disseminated in multiple chicken embryonic tissues in comparison to the viruses rLaSota and rBC(HN)^LaSota^, thus demonstrating that the HN glycoprotein determines NDV tissue tropism and contributes to viral virulence and pathogenic phenotype [130]. Similar findings have been reported for *HN* gene exchange between velogenic rJS/7/05/Ch and mesogenic rMukteswar [131], velogenic rSG10 or lentogenic rLaSota and other NDVs with different degrees of virulence [132], and mesogenic rNDVFLtag and velogenic rHerts [133] (Table 2).

**Table 2:** **Properties of recombinant NDVs harbouring F and/or HN gene exchanges**

Virus	Parent	Pathotype^*^	MDT^†^	ICPI	IVPI^†^	Reference
rBC	Beaudette C	Velogenic	62 h	1.58	nd	[130]
rBC(HN)^LaSota^		Mesogenic	72 h	1.02	nd	
rLaSota	LaSota	Lentogenic	96 h	0.00	nd	
rLaSota(HN)^BC^		Mesogenic	84 h	0.75	nd	
rMukteswar	Mukteswar	Mesogenic	46.4 h	1.44	0.16	[131]
rMukteswar(HN)^JS/7/05/Ch^		Velogenic	48 h	1.80	1.77	
rJS/7/05/Ch	JS/7/05/Ch	Velogenic	46.4 h	1.88	1.89	
rJS/7/05/Ch(HN)^Mukteswar^		Mesogenic	46.4 h	1.46	0.12	
rSG10	SG10	Velogenic	42 h	1.93	2.79	[132]
rSG10-BJHN	M	Velogenic	50.4 h	1.86	2.67	
rSG10-LaHN	L	Velogenic	57.6 h	1.76	2.38	
rSG10-HBHN	L	Velogenic	55.2 h	1.81	2.64	
rLaSota	LaSota	Lentogenic	> 90 h	0.00	0.00	
rLaSota-SGHN		Lentogenic	> 90 h	0.63	0.58	
rLaSota-BJHN		Lentogenic	> 90 h	0.51	0.41	
rLaSota-HBHN		Lentogenic	> 90 h	0.13	0.20	
rNDFL	LaSota	Lentogenic	nd	0.00	0.00	[133]
rNDFLtag	LaSota	Mesogenic	nd	1.28	0.76	
rHerts	Herts/33	Velogenic	nd	1.63	2.29	
rNDFL(F)^Herts^		Mesogenic	nd	1.31	0.41	
rNDFL(F + HN)^Herts^		Velogenic	nd	1.56	2.58	
rNDFLtag(HN)^Herts^		Mesogenic	nd	1.40	1.83	
rNDFLtag(HN)^LH^		Mesogenic	nd	1.28	1.52	
rNDFLtag(HN)^HL^		Mesogenic	nd	1.31	1.82	
rAnh	Anhinga/93	Mesogenic	88 h	0.89	nd	[134]
rTk	TkND	Velogenic	53 h	1.63	nd	
rCA	CA02	Velogenic	48 h	1.85	nd	
rAnh(HN)^Tk^	Anhinga/93	Mesogenic	84 h	1.00	nd	
rAnh(HN)^CA^		Mesogenic	72 h	0.86	nd	
rAnh(F + HN)^Tk^		Mesogenic	72 h	1.16	nd	
rAnh(F + HN)^CA^		Mesogenic	62 h	1.14	nd	
rHerts	Herts/33	Velogenic	56 h	1.54	nd	[135]
rHerts(F)^AV324^		Velogenic	52 h	1.56	nd	
rAV324	AV324/96	Lentogenic	110 h	0.10	nd	
rAV324(F)^Herts^		Lentogenic	110 h	0.00	nd	
rHerts	Herts/33	Velogenic	48 h	2.00	nd	[136]
rJS5/05	JS5/05	Velogenic	46 h	1.88	nd	
rHerts(F)^JS5/05^		Velogenic	46 h	1.88	nd	
rHerts(HN)^JS5/05^		Velogenic	42 h	1.85	nd	
rHerts(F + HN)^JS5/05^		Velogenic	46 h	1.80	nd	
rBC	Beaudette C	Velogenic	nd	1.61	nd	[137]
rBC(F)^GBT^		Velogenic	nd	1.88	nd	
rBC(HN)^GBT^		Velogenic	nd	1.71	nd	
rBC(F + HN)^GBT^		Velogenic	nd	1.80	nd	
rGBT	GB Texas	Velogenic	nd	1.91	nd	
rGBT(F)^BC^		Velogenic	nd	1.62	nd	
rGBT(HN)^BC^		Velogenic	nd	1.88	nd	
rGBT(F + HN)^BC^		Velogenic	nd	1.78	nd	
rLaSota	LaSota	Lentogenic	> 120 h	0.00	nd	[140]
rLaSota(F + HN)^SG10^		Mesogenic	79 h	1.49	nd	
rSG10	SG10	Velogenic	57 h	1.86	nd	
rSG10(F + HN)^LaSota^		Mesogenic	> 120 h	0.86	nd	

^*^Recombinant NDVs with ICPI values > 1.60 are defined as velogenic, those with ICPI values of 0.60–1.50 are mesogenic, and those with ICPI values < 0.50 are lentogenic. ^†^nd indicates that MDT and IVPI were not determined.

Notably, the ICPI values of the chimeric viruses rNDFLtag(HN)^LH^ and rNDFLtag(HN)^HL^, which separately harbour the stalk domain (aa 1–143) and the globular head domain (aa 144–561) of the rHerts HN glycoprotein, are comparable to those of rNDFLtag but lower than those of rHerts, whereas the IVPI values of these chimeric viruses are similar to that of rNDFLtag(HN)^Herts^ and much higher than that of rNDFLtag, indicating that both the stalk and globular head domains of the HN glycoprotein participate in determining NDV virulence [133]. However, replacement of the *HN* gene of a mesogenic rAnh by the corresponding genes of velogenic rTK and rCA failed to increase the virulence, pathogenic pathotype, or pathogenicity of the chimeric viruses rAnh(HN)^Tk^ and rAnh(HN)^CA^, respectively [134] (Table 2). These inconsistent results suggest that viral factors other than the HN glycoprotein are also involved in the virulence and pathogenic phenotypes of NDV.

Although the F and HN glycoproteins play collaborative roles in the replication and pathogenicity of NDV, few reports on the single replacement of the *F* gene between different NDVs exist, and the results of these studies have not always been consistent. For example, reciprocal* F* gene exchange between velogenic rHerts and lentogenic rAV324 or replacement of the *F* gene alone of velogenic rHerts by the corresponding gene of velogenic rJS5/05 did not affect the virulence phenotype of chimeric viruses [135, 136] (Table 2). However, compared with the parental virus rNDFL, the chimeric rNDFL(F)^Herts^ bearing the *F* gene of velogenic rHerts exhibited increased virulence and pathogenicity, and replacement of the *F* gene between velogenic rBC and rGBT also obviously changed the virulence phenotype of chimeric rBC(F)^GBT^ and rGBT(F)^BC^ in comparison to that of their parental viruses [133, 137] (Table 2). These results indicate that the F glycoprotein alone is insufficient to determine NDV virulence. Thus, the potential role of the F and HN glycoproteins in the determinants of NDV virulence was further assessed.

It was reported that an avirulent rLaSota with virulent Fcs (rLaSoVF) has markedly increased ICPI and IVPI values, which are similar to the pathogenicity indices of velogenic rBC; more importantly, the chimeric rLaSoVF(HN)^BC^ harbouring the *HN* gene of rBC has the same virulence phenotype as rLaSoVF and rBC, but the pathogenicity of rLaSoVF(HN)^BC^ is markedly increased compared with that of rLaSota(HN)^BC^ and rLaSota [138]. Moreover, the results of several generated chimeric viruses, in which the F and HN glycoproteins or their ectodomains were exchanged individually or together between the mesogenic rBC and avirulent rAPMV-2, revealed that the two contrasting phenotypes closely correlated with the origin of the F and HN ectodomains, which codetermined the cell fusion, tissue tropism, and virulence phenotypes of NDV and APMV-2 [139]. In addition, other researchers have demonstrated the importance of homologous F and HN glycoproteins in determining NDV virulence and pathogenicity, even though the F glycoprotein is the major individual contributor in some NDVs [134, 136, 137, 140].

Notably, because most selected NDVs may have been too divergent genetically or biologically to be compatible for gene exchange, it is unclear whether the observed effects of gene exchange reflect genuine differences in virulence determinants versus incompatibility due to excessive biological or phylogenetic divergence. Hence, more studies are needed to clarify the exact role of viral proteins in virulence through the use of NDVs with high genetic similarity and the same genotype as well as obvious differences in virulence and pathogenicity. Nevertheless, these studies still suggest that the F and HN glycoproteins codetermine the tissue tropism, virulence, and pathogenic phenotype of NDV, which greatly increases our understanding of NDV virulence and pathogenesis.

### F and HN glycoproteins contribute to NDV thermostability

Among the variety of ND vaccines, live attenuated NDVs with thermostable and avirulent properties have the advantages of being administered in drinking water, sprays and food, as well as being independent of cold chains for delivery and storage, which are widely used for ND control, especially in developing and less developed countries [141]. Most NDVs are thermolabile and lose their infectious ability when exposed to 50–55 °C for 30 min, but some thermostable NDVs can maintain HA activity and infectivity at 56 °C for more than 30 min [142]. To date, several NDVs with different genotypes and pathogenic phenotypes, including I-2 [143, 144], V4 [145], TS09-C [146], NDV4-C [147], K148/08 [148], and HR09 [149], have been reported to be thermostable. However, most of the traditionally used NDV live vaccine strains, such as B1, VG/GA, Clone-30, and LaSota, are thermolabile [150]. A previous study reported that both the HN and M proteins are associated with heat-resistant NDV [151], but the key factors determining the thermostability of NDV have not made great progress since then. In addition, studies have demonstrated that the current commercial live attenuated ND vaccines can provide ideal protection against different genotypes of NDVs, but they cannot completely block viral shedding and transmission because of the genotype mismatch of the vaccine strains with the prevalent strains [152–154]. Therefore, understanding the determinants of NDV thermostability will be helpful for the development of more thermostable and effective live ND vaccine candidates against the challenge of prevalent virulent NDVs.

At present, the reverse genetics technique has been applied to elucidate the molecular basis related to NDV thermostability. The results of viral gene exchange between the thermolabile NDV LaSota and thermostable NDV TS09-C revealed that the chimeric virus rLaSota(HN)^TS09^ bearing the HN glycoprotein of TS09-C exhibited a thermostable phenotype, and vice versa, suggesting that the HN glycoprotein is a crucial determinant of NDV thermostability [155]. In a different study, it was also found that the chimeric virus rLaSota(HN)^HR09^ harbouring the thermostable HR09 HN glycoprotein had a thermostable phenotype, and more specifically, mutations of the residues S315P and I369V in the HN glycoprotein increased the thermostability, HA titres, and NA and fusion activities of the mutant NDVs compared with those of the parental virus HR09 [156]. Consistent with these findings, another study reported that three residues, 315, 329, and 369, in the HN glycoprotein, especially mutations S315P and I369V, significantly increased the thermostability, HA, and NA activities of NDV, thus demonstrating that the HN protein is a major determinant of NDV thermostability and that residues 315 and 369 have important effects on viral thermostability [157].

Notably, the thermostability of the mutant virus rV4-HN-tr with a 39-aa truncation at the HN C-terminus was similar to that of the parental V4 strain, and the NDV-specific antibody titres and immune protection efficiency of chickens immunized with rV4-HN-tr were greater than those of V4 [51]. In addition, other studies revealed that both the HN and F glycoproteins or the P protein alone can contribute to NDV thermostability [158, 159]. The reason for these differences may be that the excessive biological or phylogenetic divergence in these gene-exchanged NDVs leads to the genuine differences in thermostability determinants, and moreover, there are still too few NDVs with similar genetic backgrounds but different heat resistances to elucidate the determinants of NDV thermostability. Therefore, more studies are needed to elucidate the association between viral proteins and NDV thermostability. Even so, these results demonstrate that the HN glycoprotein plays a major role in determining NDV thermostability and that other viral proteins also contribute to this feature. Moreover, these findings can efficiently promote the development of novel chimeric live ND vaccines with thermostable and genotype-matched features against NDV infection.

## Interaction of NDV F and HN glycoproteins with host proteins/compounds

### Interactions involved in the viral adsorption process

**Figure 4:**
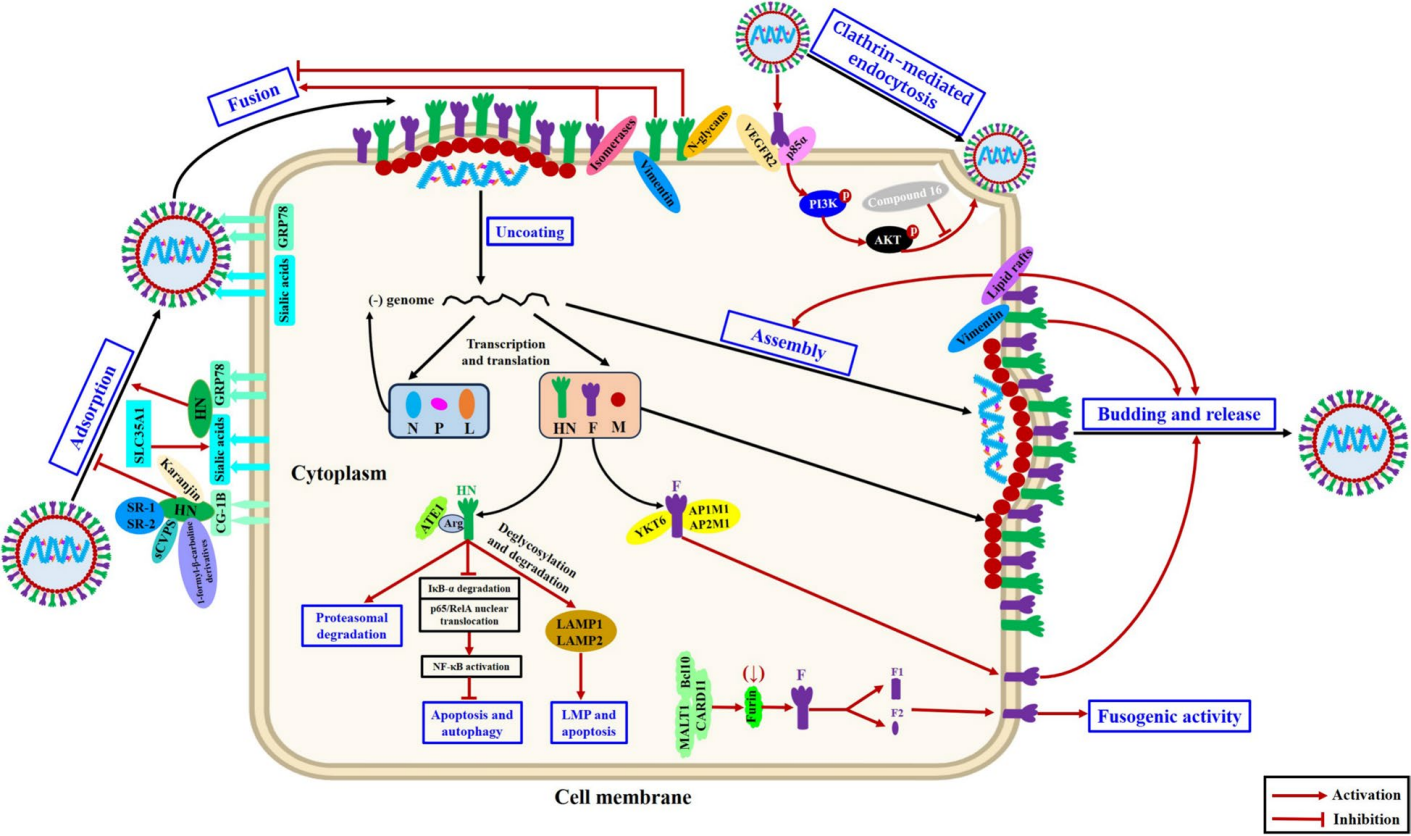
**Schematic representation illustrating the interactions of NDV F and HN glycoproteins with host proteins/compounds.** During the viral adsorption phase, binding of the HN glycoprotein to the sialic acid receptor and GRP78 promotes viral attachment to host cells, whereas the interaction of HN with host CG-1B or compounds (SR-1 and SR-2, sCVPS, a 1-formyl-β-carboline derivative, and karanjin) inhibits the viral adsorption process. In addition, either the interaction of F glycoprotein with isomerases or VEGFR2 and p85α or the interaction of HN glycoprotein with vimentin enhances the fusion and entry processes of NDV, but the addition of N-glycans targeting the HN glycoprotein prevents its interaction with the F glycoprotein and blocks viral fusion. Moreover, the formation of the CARD11‒Bcl10‒MALT1 signalosome inhibits furin expression, causing a reduction in F0 cleavage efficiency to inhibit viral fusogenic activity and viral replication and pathogenicity. Moreover, the ATE1 enzyme transfers Arg to the HN glycoprotein and drives its proteasomal degradation, and the HN glycoprotein also induces apoptosis, autophagy, or LMP by regulating NF-κB activation and the deglycosylation and degradation of LAMP1 and LAMP2, respectively, to increase viral infection and replication. Furthermore, binding of the F glycoprotein to membrane lipid rafts or the HN glycoprotein to vimentin is important for the ordered assembly and release of infectious NDV particles. In addition, the F glycoprotein interacts with AP1M1/AP2M1 or YKT6 to promote its trafficking to the cell surface and facilitate the assembly, budding, and replication capacity of NDV.

The HN glycoprotein has three major functions: (i) to attach the NDV virion to sialic acid-containing cell surface receptors; (ii) to promote the fusion activity of the F glycoprotein to allow entry of the virus into host cells; and (iii) to cleave sialic acids to release progeny virions from the surface of infected cells and prevent viral self-agglutination [99]. Among these, HN-mediated viral adsorption to host cells is the first step in determining the success of NDV infection (Figure 4). A previous study revealed gangliosides as the primary receptor and N-linked glycoproteins as the secondary receptor that are critical for NDV adsorption and entry [160]. However, subsequent studies demonstrated that gangliosides are not essential for NDV binding, fusion, or infectivity, whereas α2,3- and α2,6-linked sialic acids are required for efficient NDV attachment and entry into host cells [161, 162]. Moreover, both sialic acid receptors can initiate NDV infection in the chicken nervous system, thereby providing direct evidence for the neurotropism of NDV in chickens [162]. Recently, solute carrier family 35 member A1 (SLC35A1) was shown to be a key host factor that promotes NDV replication, as knockout of SLC35A1 decreased the expression of α2,3- and α2,6-sialic acids on the cell surface and resulted in significant decreases in NDV adsorption and internalization processes [163, 164]. In addition, another study reported the novel interaction of the host protein glucose-regulated protein 78 (GRP78) with the NDV HN glycoprotein, which plays an essential role in the adsorption stage of the NDV infection cycle [165].

Several host proteins or compounds affect the viral adsorption process by targeting the NDV HN glycoprotein (Figure 4). NDV infection can induce the sustained upregulation of chicken galectin 1B (CG-1B) in many tissues over time, and CG-1B directly binds to NDV virions to inhibit viral HA activity in vitro [166]. Further analysis revealed that the specific G4 N-glycans significantly increased the interaction between CG-1B and the HN glycoprotein, which led to a reduction in viral adsorption and replication levels [166]. In addition, some compounds, including maackiain (SR-1) and echinoisoflavanone (SR-2) [167], sulfated *Chuanmingshen violaceum* polysaccharides (sCVPS) [168], 1-formyl-β-carboline derivatives (Compounds 6, 7, and 9) [169], and karanjin [170], have been reported to reduce the viral adsorption process by directly interacting with the NDV HN glycoprotein, thus exhibiting antiviral activity. Notably, the R197, H199, R363, and R523 residues within the NDV HN glycoprotein are the main binding sites for sCVPS, and binding to R363 is especially effective at masking sialic acid binding sites, which provides valuable information for the development of efficient antiviral strategies against NDV and related paramyxoviruses [168]. Moreover, computational exploration and molecular dynamic simulation in a recent study revealed several antiviral agents targeting the NDV HN glycoprotein, but the mechanism of action of these compounds remains to be clarified [171]. Taken together, these results indicate that the design of receptor- or HN-based antiviral drugs is an efficient antiviral strategy to prevent viral adsorption and infection.

### Interactions involved in viral fusion and entry processes

To date, interactions of host proteins or compounds with the F or HN glycoprotein have been reported to affect the fusion and entry processes of NDV (Figure 4). A previous study revealed that the addition of N-glycans to the stalk domain (residues 69 and 77) of the NDV HN glycoprotein prevents its interaction with the F glycoprotein and blocks viral fusion [78]. Moreover, the interaction of F glycoprotein with cellular isomerases is increased after HN binds to cell surface receptors, which causes a reduction in disulfide bonds in the NDV F glycoprotein to produce free thiols that are required for fusion at very early stages during the onset of fusion [172]. In addition, a recent study reported that vimentin exhibited a much stronger interaction with the HN glycoprotein of mesogenic NDV than with that of velogenic NDV, but it participated in multiple aspects (including viral internalization, fusion, and release) of the lifecycle of velogenic NDV rather than that of mesogenic NDV in chicken macrophages [173] (Figure 4), thus revealing the pivotal role of the HN–vimentin interaction in velogenic NDV infection.

In addition to receptor binding and membrane fusion, researchers have focused on different endocytic routes involved in the NDV entry process [174]. Three main endocytic pathways, clathrin-mediated endocytosis [175], dynamin- and caveola-mediated endocytosis [176–178], and macropinocytosis [179, 180], have been shown to mediate the entry of NDV into cells. One recent study revealed that NDV F glycoprotein and vascular endothelial growth factor receptor 2 (VEGFR2), along with the phosphatidyl-inositol 3-kinase (PI3K) subunit p85α, interact and colocalize at the cell membrane, promoting the phosphorylation of PI3K and AKT to activate the PI3K/AKT signalling pathway and induce clathrin-mediated endocytosis to establish successful viral infection [175] (Figure 4). Notably, another study revealed that Compound 16, one of the canthin-6-one analogues, presented the strongest anti-NDV activity by suppressing the AKT pathway to inhibit the entry of NDV [181]. Moreover, Compound 16 treatment also inhibited the NDV-activated ERK pathway and promoted the expression of interferon-related genes, thereby underscoring the potential value of canthin-6-one analogues as candidate antiviral agents for NDV and related paramyxoviruses [181]. Accordingly, these findings deepen our understanding of the molecular mechanism of NDV fusion and entry into cells and reveal novel targets for antiviral strategies supporting infection prevention and control measures.

### Interactions involved in the viral intracellular replication process

In addition to the role of the F and HN glycoproteins in the adsorption, fusion, and entry processes of NDV, numerous studies have confirmed that the interaction of host proteins with the F or HN glycoprotein regulates the intracellular replication of NDV in a variety of ways (Figure 4). It has been reported that the cellular protease furin is responsible for cleaving the NDV F_0_ protein to form two disulfide-linked subunits (F1 + F2) in cells, which catalyse membrane fusion to juxtapose the virus‒cell membrane. However, the expression level of furin can be regulated by cellular signalling proteins in some tumour cells [182, 183]. One recent study demonstrated that the overexpression of caspase recruitment domain-containing protein 11 (CARD11) significantly reduced velogenic NDV-induced syncytium formation without altering the fusogenic property of progeny virions, and this phenomenon was also observed in viral F- and HN-coexpressing chicken fibroblasts [184]. Further analysis revealed that the formation of the CARD11‒Bcl10‒MALT1 signalosome inhibits the expression of furin during NDV infection, thus reducing the cleavage efficiency of the F_0_ protein and inhibiting viral fusogenic activity as well as viral replication and pathogenicity [184] (Figure 4).

In addition, NDV infection induces increased levels of the arginyltransferase 1 (ATE1) enzyme, which transfers an Arg residuec to the N-terminus of the HN glycoprotein and ultimately induces its proteasomal degradation to impair viral replication [185] (Figure 4). Moreover, another study revealed a novel function of the HN glycoprotein in increasing the infection and replication of velogenic NDV, as evidenced by the suppression of IκB-α degradation, p65/RelA nuclear translocation, and NF-κB activation by the velogenic NDV HN glycoprotein, which eventually resulted in increased apoptosis and autophagy to promote viral infection and replication [186] (Figure 4). Furthermore, NDV infection strongly triggers lysosomal membrane permeabilization (LMP) and leads to mitochondria-dependent apoptosis, and importantly, the sialidase activity of the HN glycoprotein induces the deglycosylation and degradation of lysosome-associated membrane protein 1 (LAMP1) and LAMP2 and contributes to NDV-induced LMP and apoptosis, ultimately increasing viral replication in tumour and avian cells [187] (Figure 4). Therefore, these findings reveal the novel functions of F and HN glycoproteins in the replication and pathogenesis of NDV and offer valuable insights for the development of antiviral drugs that target viral glycoproteins or interacting host proteins to block NDV replication.

### Interactions involved in viral assembly, budding, and release processes

In recent years, an increasing number of studies have confirmed that F and HN glycoproteins participate in the assembly, budding, and release processes of progeny NDV virions through interactions with host proteins (Figure 4). The CT domain of F glycoprotein is tightly associated with membrane lipid rafts, which are important for the ordered assembly and release of infectious NDV particles [188, 189]. Moreover, cholesterol and membrane lipid rafts are required for the incorporation of functional HN‒F glycoprotein-containing complexes into NDV virions but are not necessary for the stability of preformed HN‒F complexes once they are assembled into virions [190]. In addition, two other studies reported that the YLMY tyrosine residues within the CT domain of the F glycoprotein regulate its cell surface expression to modulate viral budding and pathogenicity [32], and in particular, the YLMY motif mediates the interaction of the F glycoprotein with the host adaptor protein (AP) complexes AP1M1 and AP2M1, which directs F glycoprotein transportation to the cell surface and promotes its ability to affect the viral budding process [33].

Moreover, one recent study revealed that NDV infection significantly upregulated the expression of the synaptobrevin homologue YKT6, which efficiently protected the F glycoprotein from degradation by the ubiquitin‒proteasome system and facilitated the trafficking of the F protein from the ER‒ERGIC‒Golgi pathway to the cell surface, thereby increasing the assembly, budding, and replication capacity of NDV [191]. In addition to the known function of vimentin in affecting viral fusion, it plays a crucial role in promoting the release of NDV virions, as siRNA-mediated knockdown of vimentin clearly reduced viral HA titres in virus-infected cells [174]. Taken together, these interaction studies deepen our understanding of the replication and pathogenicity of NDV, but more exploration is needed to investigate the potential role of F/HN-host protein interactions in the assembly, budding, and release processes of NDV.

## Future prospects

Paramyxovirus members, including PIV5, NiV, hPIV3, CDV, and NDV, are notorious for their devastating infections and substantial economic burdens in humans and animals. Although different paramyxoviruses share a similar cohort of genes but with distinct differences in gene and protein homologies, structural and functional properties of these viral proteins can still be inferred from each other. To date, studies on the virulence, infection, replication, and pathogenicity of the NDV F and HN glycoproteins are abundant, but there is a noticeable imbalance in the study of the precise structures of these proteins. For instance, the current understanding of the structural features of the uncleaved and cleaved metastable F trimer, the prefusion F trimer ectodomain conformation, the conformational rearrangement of the HN/F interaction as fusion proceeds, the tetrameric HN in complex with the receptor, and the F trimer in complex with HN-stalk is still less thorough than for those of other paramyxoviruses and other NDV structural proteins. In addition, recent studies have focused on resolving the structures of neutralizing antibodies targeting the paramyxovirus (represented by hPIV3, NiV, HeV, and MeV) F or HN/G glycoproteins and developing broad-spectrum antibodies against a variety of paramyxovirus infections [46, 83, 192, 193], but limited attention has been given to the structural complex between the antibodies and the NDV F or HN glycoproteins, which needs further exploration.

Numerous studies have indicated that knowledge of the structure and function of NDV glycoproteins may provide a fundamental basis for the discovery of novel antiviral drugs. Therefore, the development of experimental anti-NDV natural compounds or drug databases is helpful for the development of efficient inhibitors for other paramyxoviruses. There is evidence that NDV-HN and hPIVs-HN share a high degree of similarity in catalytic site structures, and the design and synthesis of sialic acid derivatives as inhibitors of NDV-HN can provide efficient NA inhibitory activity for NDV-HN and hPIVs-HN [194]. In recent years, cryo-electron microscopy has been successfully applied to resolve the precise structures of F and/or HN glycoprotein monomers and complexes of several paramyxoviruses (such as MuV, hPIV3, and NiV), which has provided valuable reference information for the structural study of other paramyxovirus (including NDV) F and HN glycoproteins. More importantly, structure-based subunit vaccine design, including the engineering of the paramyxovirus F glycoprotein to stabilize its prefusion conformation or a chimeric immunogen composed of prefusion F linked to the viral HN glycoprotein, can lead to the development of promising primary series or booster vaccine candidates for various paramyxoviruses. Collectively, the resolution of F/HN-related structures can increase our understanding of the mechanism of viral membrane fusion and entry into host cells and furnish a blueprint for next-generation structure-based vaccines, broad-spectrum antibodies and antiviral drugs against paramyxovirus infections.

The F and HN glycoproteins are major determinants of the virulence, pathogenic phenotype, and thermostability of NDV, but the results of some studies do not always agree, possibly because of the excessive biological or phylogenetic divergence between the selected NDVs. Therefore, NDVs with high genetic similarity and obvious differences in virulence and pathogenic phenotype should be preferentially considered for subsequent relevant studies. There are some good examples, such as velogenic NDV-Blackbird and lentogenic NDV-Dove, which belong to genotype IX and have genomes that are 99.9% similar, with only five inconsistent aa sites in the HN protein, with residues L110 and R116 found to principally determine the difference in virulence between these NDVs [195]. Moreover, a genotype III mesogenic vaccine strain, Mukteswar, and its evolved velogenic NDV JS/7/05/Ch exhibit major aa mutations in the HN protein, of which A494D and E495K mutations are responsible for the increased virulence and pathogenicity of velogenic genotype III NDV [196]. In addition, 15 virulent strains isolated from southern Angola are grouped within genotype VII.2 and classified into thermostable and thermolabile NDVs [197], providing the original materials for systematically exploring the major determinants of NDV thermostability. Thus, these NDVs will be helpful for elucidating the exact role of viral proteins in the virulence, replication, and pathogenicity of NDV and developing more thermostable and efficient genotype-matched live ND vaccines in the future.

Beyond that, the virus‒host protein interplay generally regulates various aspects of NDV; however, investigations into F/HN–host protein interactions remain relatively limited. These limitations highlight the need for continued exploration of the potential roles of F and HN glycoproteins in the NDV lifecycle and pathogenesis. In addition to traditional coimmunoprecipitation and pull-down assays, genome-wide CRISPR/Cas9 library screening has become advantageous for identifying essential host factors for the replication of intracellular pathogens. By this method, a variety of novel host factors, such as SLC35A2, SLC35A1, and β-1,4-N-acetyl-galactosaminyltransferase 2 (β4GALNT2), have been found to affect the fusion and adsorption events of several paramyxoviruses [163, 164, 198]. Notably, several compounds have been demonstrated to exert efficient antiviral activity against NDV and related paramyxoviruses by targeting viral glycoproteins, which represent suitable scaffolds for the discovery and design of novel antiviral drugs to combat the infection of multiple paramyxoviruses by means of modern molecular biology techniques.

## Conclusion

The F and HN glycoproteins have attracted much attention because of their extensive and essential functions in terms of NDV virulence, pathogenic phenotype, thermostability, infection, replication, and pathogenicity over the past 50 years. Although considerable work has been carried out to elucidate both the unique and common functions of NDV glycoproteins, research on the structure and function of these two proteins is not as in depth as that of most paramyxoviruses. With respect to the unsolved questions discussed in this review, studies involving analysis of the protein‒protein interactome, single-particle cryo-electron microscopy, genome-wide CRISPR/Cas9 library screening, and computational structural biology will provide novel insights into the enigmatic replication and pathogenicity mechanism of NDV. Therefore, more insights into the structure and function of the F and HN glycoproteins can aid in understanding the replication and pathogenesis of NDV and related paramyxoviruses and provide a theoretical basis for the future development of efficient treatment strategies.

## Data Availability

Data sharing is not applicable to this article, as no new data were created or analysed in this study.
